# Are We Punishing the Right Man?

**Published:** 1899-08

**Authors:** J. B. Hodgkin

**Affiliations:** Washington, D. C.


### ARE WE PUNISHING THE RIGHT MAN?

BY DR. J. B. HODGKIN, WASHINGTON, D. C.

No one who observes the trend of events but must feel that all who are engaged in the supervision of dental education, whether in a professional way or as members of examining boards, are really in earnest as to the matter of doing what they profess, and wish most earnestly to forward in every way the interests of dental education. If there are doubts as to methods, they seem doubts as to methods only, and not as to the need for advanced teaching, or of the desire of all engaged in the work to conspire to the fulfilment of the best aims and needs of the profession. That the ways and means now employed are not the very best probably all, even those who have worked hardest in this direction, are willing to admit; and we, though not seeing the clearest and best light now, are focussing all glasses on the horizon, in the hope that the light, still too dim for our purpose, may grow more luminous, and that soon.

But while this is true, and while we who are teaching and those whose function it is to legally supervise afterwards the work done in the colleges before it is passed as "good" see partly the effect of the work and the supervision, still I take it that a little earnest quarrelling over the methods used by both parties may not be useless; for out of wrangling comes clear thought, and if argument does nothing to convince the arguers, it does convince the bystander.

I have headed this with the words, "Are we punishing the right man?" a question involving the thought that some one is being wronged. I wish to state, as briefly as I may, what the wrong done is, to whom and by whom it is done, and the remedy, if such can be found and applied.

Our college session is now three years, each in separate years, and each session of seven months. This, subtracting the holidays, may make a full six months, or, altogether, about eighteen months in college. Of these months, the six of the first year are devoted to elementary studies, which I need not enumerate; the others, supposedly, to the real study of the science of dentistry as applied to work. The theory of the schools, and the arrangement of the classes is in accord with the idea that it will take at least three years for the average man to acquire such a knowledge of dentistry, with the means furnished by the colleges, as will fit him for practice. Perhaps this may be a fact, as almost all the schools of the country have accepted this schedule as necessary, and are run on the idea that unless the average man has at least three years of pretty close application he will not be fitted for practice.

This idea has been so persistently drilled into the dental (public) mind as to be accepted as a truism, and being accepted as vox populi, becomes vox Dei, and settled upon as an ultimate truth.

But I, with that freedom which I have the right to claim, ask, Is this true? and if so, is there no fault to be found in the arrangement? As to whether the first part be true I will not stop to discuss; the fact may be that it *does* take three years on the average to make a good dentist; but is it a fact that because it takes this three years *for some,* should all, whether capable of taking in the course in one year, two years, or three, be compelled to work together all the three years? Now, as a matter of fact, all who have had the most superficial notice in the matter of education know that there are different degrees of apprehension with students. It would be foolish to do more than state this, and foolish in you to deny it. You know men, I know men, who learned more of dentistry, more of anatomy, more of mathematics, more of music, in one week than another would learn in one month. So obvious is this that I will no more than mention it. What we do in our schools is to make this ready wit, this alert brain, this comprehensive and apprehensive mind, *drudge with the dull fellow who can only, by the most patient toil,* make his average in the three full years of study. The answer I get when I assert that this is unjust, is that the injustice of shortening the term, or of allowing the student the choice of trying for graduation sooner, is less than the occasional injury inflicted on the stronger pupil. The involved idea is that had the colleges any power in the matter of graduation in less than the three years' limit, its abuse, even if seldom attempted, would be a worse calamity than the abuse of keeping the proficient man alongside the dull one for the full term.

Here is the gist of the whole matter; here the sin of which I complain. I contend that no college, no board of examiners, no board of regents, or other power, has a moral right to harness a bright man to a dull one and say to the great mind: "True, you are strongly capable, and the fellow with you feebly capable; you could easily master this curriculum in twelve months or less, and he can at best do it only within three years, but public opinion says (and we must bow to it), that if any other course be allowed, the incompetent man may slip through and injury be done."

But, gentlemen, I wish to say that none of us has a right to punish the wrong man. None of us has the right to inflict an injustice on one man that the chance of injustice to another, or to others, may be less. The law has a maxim that it is better that ninety-nine guilty men escape rather than one innocent man be found guilty. And to this maxim the common sense of mankind assents. How, then, can we, who are educating professional men, say to them, "We know you are not all equally bright, and that some of you could acquire all that any board of examiners, or any college examiners, could ask inside of one year, but we are afraid that if we allow one of you to do this, that some other one may possibly slip by and escape with a diploma to the injury of the profession. The question with us is that we must not let any man graduate until entirely fit; we accept the dictum of the boards that this takes three years for the average man, and so you must take your place with the slow and keep pace with the dull, else we may not be able to discriminate. In a word, gentlemen, we are not trusted by the boards in this matter, and so you must take the consequences. The truth is your brightness is just now your misfortune."

This is just what happens right here in our Washington (D. C.) High Schools, and in the graded schools as well. Everybody who knows anything about the system in our schools knows that there are children who could and do master the curriculum of the classes in half the time that others take. It is simply a question of capacity. I have a boy in the high school here who will do his algebraic equations and talk over his shoulder with the other children at the same time, while another has to concentrate all his energies on such work, and seeks the quietest solitude that he may painfully work out what the other does as mere play. To tie down this strong-brained boy and compel him to keep pace with the slow plodder is a manifest injustice, yet our schools have no other than this machine way of instruction, and possibly no other way is feasible in our day.

But if I "show you a more excellent way," what then? There is such a way, and a school—possibly the most successful school—in this country for years, and successful still, has such a method. I refer to the University of Virginia, than which there has been no more thorough and efficient teacher of young men for three-fourths of a century. When I tell you that not more than ten years ago nine men graduating from this university took the examination for surgeon in the United States navy, and that of the nine, eight passed this most trying ordeal, and that the same year nine graduates from Harvard took the same examination, and eight out of the nine Harvard men failed, you will appreciate something of the high standard of this University, founded by Thomas Jefferson, and from which have graduated many of the brightest lights of our land.

The course at this school is, as one of its graduates expressed it to me, "tough." It is nine months of solid work, nine hours a day, in school, every day except Sunday, and one day's holiday at Christmas. The candidate for entrance has, of course, to pass a preliminary examination, and if this is satisfactory he is admitted to the medical school (for it is of the medical I am thinking,—there is no dental department), and for four and a half months follows the prescribed course of study. At this time he is allowed to try for the middle examination, and if successful in this he is eligible for graduation, providing he can pass the "final." At the final he is tested, and some *do* graduate in one session. I have known, I think, three to do this; probably more have done so, but of this I am not certain. I knew of one class of seventy-three medical students, of whom four graduated!

Now, as I have said, these men try for such examination as the navy or the army, and I am told they are quite as successful in the army examinations as in the navy. Yet this is a college which allows students to graduate in one session,—*if they can.* If one contrasts this with the other method of harnessing down men who could graduate in such a school in one session to a course of three, it will be seen that the crime I have spoken of in the title to this essay is a real one, and an unjustifiable punishment is inflicted on the innocent but competent pupil. If the University of Virginia can do this and keep clean hands, any school can and should. If men can show their ability, after a single year in such a school as this, to pass such examinations as those required by the army and navy, it is a shame to keep them longer.

The capable student says—he and his friends—the long sessions are made for the *benefit of the college;* the three terms made for the *gain of the teachers.* Here extremes meet, for in forcing on the colleges a three years' course the Examiners have, unwittingly, possibly, put money in the pockets of the professors.

For every evil there should be a remedy, even for the social evil. The one I suggest may be far off and apparently out of grasp, but it is, at all events, simple and radical.

We spend millions annually in education. How much is given away in this way who can tell? What I suggest is as simple as can be. It is to take from the colleges all power over the final examination. Let this be the function of a board provided for by act of Congress. I will presently explain this more fully.

Now, let this board do all the work of the "finals," take all the responsibility, grant the diplomas, in the name of and for the college, and let this examination and its results be the thing by which the student is to pass muster outside. The function of the State Boards to be simply that of endorsing the action of the examiners in chief, and they (the State Boards) electing from among their number the examining board; these in turn to be confirmed by Congress, just as other judiciary appointments are.

It will be objected that the State Boards will not agree to this, and yet, if they will consider, they will really have more power than ever, since they will virtually control the schools. And if objection is made that the States may not agree to such arrangement of the matter and of the practical annulment of the State laws, it is sufficient answer that each State law is about what the best dentists of the State ask for; and can a better plan be suggested for the unification of all the State laws than for a board, nominated by the National Examiners, to formulate as perfect a system of examinations as that used by the army or navy for its candidates.

Of course, we know that there resides no power in Congress to legislate after this fashion for the States, but it is not at all impossible for men who are really *in earnest* in the matter of education to agree to lay aside all personal views and *agree upon something.* I am of the opinion that if the National Board of Dental Examiners were to meet in good faith a board from the colleges, and agree to a set of questions to which all who apply for graduation must answer, that the two could hardly fail to come to the best sort of agreement in the matter. Now, if the nominations made for the Board of Examiners for the country were made free from political or other bias, and Congress were asked to ratify this selection, and then the boards for the States cordially agree to accept these as final, how great would be the improvement on what we have now!

The State laws, of course, would have to be modified, but this, I take it, should not be impossible if the Examiners were really in earnest. The list of questions would have to be made up from year to year by the Board, and copies furnished the colleges as a basis of work; or, if it were better, the teachers might furnish lists of their own questions to the Board, with the monthly examinations (written) of the classes.

It would only be necessary for Congress, which appropriates millions a year on so-called education among other classes than dentists, to make such appropriation as may be needed; or the States might make a special tax for this purpose, it being possible that this last might be the better plan.

What is needed, of course, is practical agreement among those educating and those controlling education in our midst. Some plan such as I suggest need not necessarily hamper the work or the individuality of any college or any teacher. It would leave all free to teach as each thought best. But it would leave the results to be tested by outside parties, parties uninfluenced by interest or by friendship. It would make the matter of graduation one of merit solely, and would place the whole matter where it rightly belongs,—out of the control of the schools; and best of all, possibly, it would rid the graduate of that subsidiary and, to him, degrading State Board Examination.

The difficulty which appears at first sight of the colleges closing about the same time of year, and of piling on the Examiners an amount of work impossible of performance, may be met by providing that the Board be allowed six months or less for the "final," and that they travel from State to State for this purpose, the college students meeting them at convenient points, just as at present they meet the State Boards. Report of the Examiners to be made direct to the Deans of the colleges, or otherwise, as may be thought best. If this be thought a hardship on the student and prospective graduate, let the State Board allow him a provisional license to practise in any State until the report of the National Examiners is made.

Of course, this implies the constitution of a board whose duties may be so large as to take most of their time. But it need not; and, indeed, it seems tolerably certain that such a board might well do this work in, say, the three summer months, and not injure the plan at all by this. The student need not suffer if he is licensed meantime, and the profession could not suffer, inasmuch as the harm the student might do would be self-limiting.

In conclusion, do not jeer at this as the scheme of a dreamer, but think of it as the suggestion of one who has seen for years the unhappy results of the friction between college faculties and State boards, and who has seen and felt the "personal equation" which must show itself at the end of the session, when the "boys" who sat on the benches during lectures are now appealing to the sympathies of the teacher.

Let the "one session idea" dwell in your minds a little. Let you but think of the injustice to your own son of keeping him, a bright, intelligent lad, harnessed to a dullard for three long years, when you know that he is as receptive as a sensitized photographic plate, and think how the iron-clad three-years' rule makes this necessary in multitudes of cases in every year in college classes, and how you are punishing the bright man for being bright, and how you are encouraging dulness in being dull, so it be persistent, and I think you may agree at least to think over this subject with me.

To summarize:

1. A single session, of nine months.

2. An intermediate examination for admission to the graduating class.

3. Opportunity for all to take the one course, and graduate.

4. Teachers to be teachers only, and to have no part in decision of award of diplomas.

5. A board of examiners to be selected by the National Association of Examiners, sitting in conjunction with college board, or alone, these to be confirmed by act of Congress, and paid by Congress, as public educators.

6. The decisions of this board to exempt those who come before it from any "retrial of the case" before the State Boards.

7. A provisional license for the applicant for a diploma until he has passed the "final."

Some provision might be made for the retrial of cases, and of course rejected applicants could return to college for another term.

